# Experiments on the Gaseous Oxide of Azote, by a Society of Amateurs, at Toulouse

**Published:** 1807-01

**Authors:** M. P. Dispan

**Affiliations:** Professor of Chemistry in the College of that City


### *Experiments on the Gaseous Oxide of Azote, by a Society of Amateurs, at Toulouse; communicated by* M. P. DISPAN, Professor of Chemistry in the College of that City, to the Editors of the Annales de Chimie.

THE different, and even contradictory results, which have been published, from time to time, respecting the

 effects effects produced by the respiration of the gaseous oxide of azote, led to the following experiments, which were performed on more than a dozen persons, and in some cases repeated two or three times on the same individual. The sensations experienced by each, were carefully noted down at the moment, and the following account is drawn from these memorandums.

The nitrate of ammonia employed in these experiments, was indistinctly crystallized, but perfectly neutral. Its taste was extremely pungent, with a slight odour. It had been formed by saturating very pure nitric acid with ammoniacal gas obtained from the distillation of sal ammoniac, with the common potash of the shops.

About 1545 grains of this salt were put into a small retort, and placed on a sand-bath; the salt melted and boiled for a short time without yielding any gas, but at length the retort became filled with a white vapour, which however quickly disappeared; after which the gas was rapidly disengaged, and received in bladders.

The same process was performed on a larger scale 10 oz. troy of the salt being employed, from which a sufficient quantity of gas was obtained to fill eight bladders. In this last case, a red vapour arose within the retort as it began to cool, which was ascertained by experiment to contain no nitrous gas.

The gaseous oxide of azote, when tasted or inhaled, is generally allowed to possess a strong saccharine taste, which frequently remains on the organs during the whole day after receiving it. M. Dispan observed in it an after-taste of nitre, but acknowledges that it was the last collected gas which he tasted. Another gentleman belonging to the Society, perhaps under a similar impression, says he perceived in it an astringent quality.

In these experiments the gas was respired by means of a bladder furnished with a stop-cock, which was applied to the mouth; the nostrils being closed, and the lungs emptied as much as possible.

The first person upon whom the experiment was tried, swooned at the third inspiration, and remained senseless about five minutes, when he recovered, but with a sensation of great lassitude and fatigue. He recollected to have experienced only a sudden faintness, attended with a tingling at the temples.

The second, M. de M. observed a saccharine astringent taste; he experienced a sense of great dilatation, accompanied with heat in the breast; his veins swelled, his pulse pulse quickened, and he became vertiginous. But he thought he could have borne a stronger dose; the bladder not being large enough for his lungs.

The third experienced a saccharine taste on the first inspiration, but was insensible to those which succeeded; his lungs were forcibly dilated with great heat. When the bladder was removed, he appeared very comfortable, but could not refrain from violent bursts of laughter.

The fourth individual subjected to this experiment experienced the same saccharine taste as those who preceded him, and he retained the impression from ten o'clock in the morning till past midnight. His legs trembled under him, and he became frequently vertiginous during the remainder of the day.

The fifth perceived the same saccharine taste. On resigning the bladder he was seized with dizziness, succeeded by a sensation of great pleasure throughout the body; he felt likewise a sense of great weakness in his lower extremities.

The sixth subject of this experiment perceived the saccharine taste throughout the remainder of the day; his ears tingled, his legs tottered, his stomach was oppressed, and altogether his sensations were rather painful than agreeable.

With the view of ascertaining if the mode of breathing from a bladder might produce any influence on the foregoing results, the same individuals were requested to inspire atmospheric air in the same manner. They were all fatigued by it, but nothing more. The bladders were next filled with oxygen gas; and the only difference between it and common air was, that it produced an increase of heat in the lungs. The singular effects, therefore, which are described above, can only be attributed to the gaseous oxide of azote.

At a subsequent meeting of the Society, other and more extensive experiments were entered on. 27 ½ ounces troy of nitrate of ammonia, prepared as before, were put into a retort, with its neck fitted to a double-bodied receiver; from whence, by means of a tube, the gas passed into a vessel inverted over water. The retort was placed on a sand-bath. As soon as the heat affected the retort, the salt melted; and nearly at the same moment sparkling vapours arose in the retort, but in a very small quantity. The air which the heat expelled from the vessels had a nitrous odour; but this, as well as the vapours, gradually diminished; as the process continued, they disappeared

 however however altogether, and were succeeded by a lively smell of Prussic acid. At length the retort became filled with white vapours, and the gaseous oxide of azote began to pass over. The disengagement soon became so abundant, that it was judged proper to withdraw the fire; but afterwards, on replacing the coals, the gas which had diminished in the interval was again so rapidly evolved, that the luting of the vessels began to give way; yet, notwithstanding the loss thus occasioned, the disengagement continued extremely rapid in the receiver for at least a quarter of an hour.

M. Dispan supposes, that an explosion was only prevented in this case by the luting having given way. He next proceeds to state the effects which were produced by the respiration of the gas. Twelve persons were subjected to the experiment, and on many it was repeated. Most of them had inhaled the gas on the former trials, when two out of seven experienced pleasing sensations, but on the present occasion not one felt pleasure; on the contrary, they all felt pain, and many of them suffered severely.— One person in particular, stamped with his foot during the whole time of breathing the gas. When the bladder was removed, he recovered from the profound stupor into which he had been thrown, and complained of a pain in the back part of his head, as if he had received a violent blow from a dagger; his sensations were so painful that he could not be prevailed on to make a second trial. The other individuals were in general affected with vertigo and dizziness, succeeded in some by involuntary convulsive fits of laughter.

M. Dispan's description of the effect of this gas upon himself is thus recorded. "At the first inspiration," says he, "I emptied the bladder, on which I felt a saccharine taste in my mouth, and my lungs immediately became in-inflated. I emptied and filled them again; but upon the third attempt, my ears were affected with a tingling noise, and I dropped the bladder. I did not, however, wholly lose my consciousness, but remained in a kind of benumbed astonishment, rolling my eyes about at random. I was then suddenly seized with convulsive fits of laughter, such as I had never in my life experienced; in a few seconds, however, this propensity to laugh suddenly ceased, and I no longer felt any unpleasant symptoms."

Two others, on whom the gas was tried, experienced only a convulsive motion of some of the muscles of the face; face; but were, in the course of the day, attacked with violent diarrhœa.

M. Dispan is of opinion, from every thing he observed, that it will be extremely difficult to reduce the effects of gaseous oxide of azote to any general system, as they vary so considerably on different individuals: and, what is more singular, even on the same person.

This paper is concluded with an account of some experiments made by M. D. in order to ascertain the effects of gaseous oxide of azote upon animals. He placed a greenfinch in a vessel of sufficient dimensions, and filled it with the gas in question. At first, the bird seemed to suffer no inconvenience, but he soon gradually closed his eyes, and dropped gently on his side, as if asleep. On being restored to the pure air, he resumed his feet, without however attempting to fly away. About an hour afterwards, he was subjected to a second trial, and having been suffered to remain longer in the vessel, he was taken out quite dead. M. Dispan thinks it very remarkable, that the bird should make no effort to escape, or that he should manifest no convulsive symptoms, such as take place in experiments with other gases.

